# Temporal Bone Cholesteatoma Presenting With Chronic Facial Nerve Palsy: A Case Report With Radiological Correlation

**DOI:** 10.7759/cureus.106458

**Published:** 2026-04-05

**Authors:** Krishna Mohan Kaimal, Rakesh Gali, Joshua Lauder

**Affiliations:** 1 Otolaryngology - Head and Neck Surgery, Royal Blackburn Hospital, Blackburn, GBR; 2 Radiology, Royal Blackburn Hospital, Blackburn, GBR

**Keywords:** case report, cholesteatoma, diffusion-weighted imaging, facial nerve palsy, skull base surgery, subtotal petrosectomy, temporal bone

## Abstract

Temporal bone cholesteatoma may present atypically, including cases that lead to isolated facial nerve palsy. We report a 72-year-old man with an eight-month history of stable right-sided lower motor neuron facial weakness. Examination demonstrated House-Brackmann grade III facial nerve palsy. Imaging revealed a lesion involving the right mastoid and jugular bulb with bony erosion and diffusion restriction on MRI, consistent with cholesteatoma. The case was discussed at a regional skull base multidisciplinary team meeting, and subtotal petrosectomy with blind sac closure was planned. This case highlights the importance of imaging in persistent facial palsy and multidisciplinary decision-making in advanced temporal bone disease.

## Introduction

Cholesteatoma is a keratinizing squamous epithelial lesion of the temporal bone that can lead to progressive bony destruction and significant complications if untreated [1,2]. It most commonly arises in the middle ear and mastoid but may extend to involve adjacent structures, including the facial nerve canal. Facial nerve palsy is an uncommon but recognized complication, typically associated with advanced disease and bony erosion of the fallopian canal [3,4].

The temporal bone houses the facial nerve within the fallopian canal, making it vulnerable to compression or erosion by expanding cholesteatoma. Early diagnosis is therefore essential to prevent irreversible neurological deficit. Radiological imaging plays a central role in assessment, with diffusion-weighted magnetic resonance imaging being particularly useful because it can demonstrate restricted diffusion due to keratin content, thereby aiding differentiation from other lesions [5,6].

We present a case of cholesteatoma presenting with isolated facial nerve palsy, highlighting the importance of imaging in diagnosis and the role of multidisciplinary management in guiding definitive surgical treatment.

## Case presentation

A 72-year-old man presented with an approximately eight-month history of right-sided facial weakness, with symptom onset in February 2025. The weakness had remained stable without progression or improvement. Notably, facial weakness was the sole presenting symptom, with no preceding or concurrent otological complaints such as otorrhea, otalgia, or vertigo. He had a history of tympanoplasty during adolescence.

Clinical examination demonstrated a right lower motor neuron facial nerve palsy, graded as House-Brackmann grade III [7]. He was able to close his eye completely with effort. Otoscopy revealed intact tympanic membranes bilaterally. Examination of the remaining cranial nerves was normal. Neck and parotid examinations were unremarkable. There was no relevant drug history, including no use of ototoxic medications.

Pure-tone audiometry demonstrated a moderate-to-severe mixed hearing loss in the right ear. Bone conduction thresholds were 10 dB at 500 Hz, 15 dB at 1 kHz, 60 dB at 2 kHz, and 40 dB at 4 kHz, with an average air-bone gap of approximately 20 dB, confirming a superimposed conductive component. The left ear showed age-related high-frequency sensorineural hearing loss consistent with presbycusis.

**Figure 1:**
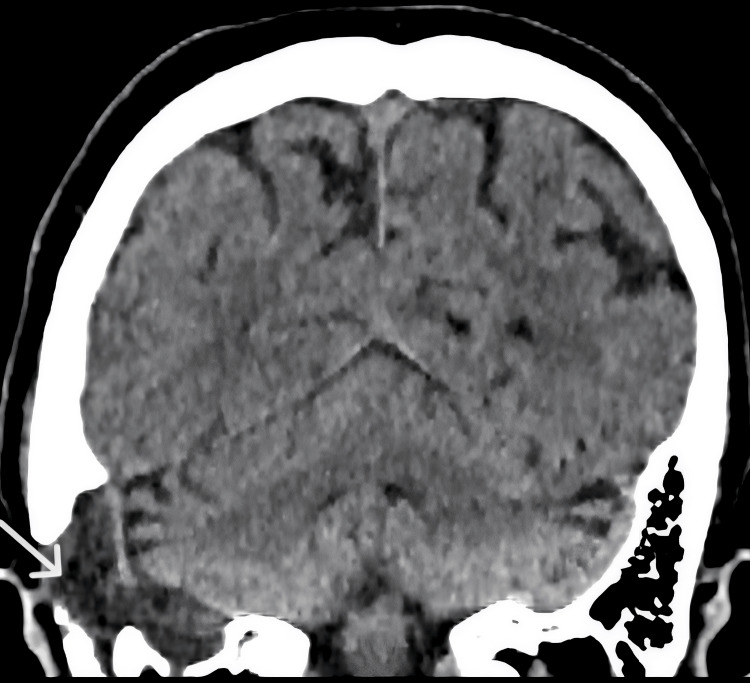
Unenhanced coronal CT image demonstrating a low-density lesion involving the right mastoid (white arrow) with associated bony erosion. The lesion is seen in proximity to the mastoid segment of the facial nerve canal

Unenhanced CT of the temporal bones demonstrated a low-attenuation lesion involving the right mastoid with associated bony erosion and effacement of the mastoid segment of the facial nerve canal (Figure 1).

**Figure 2:**
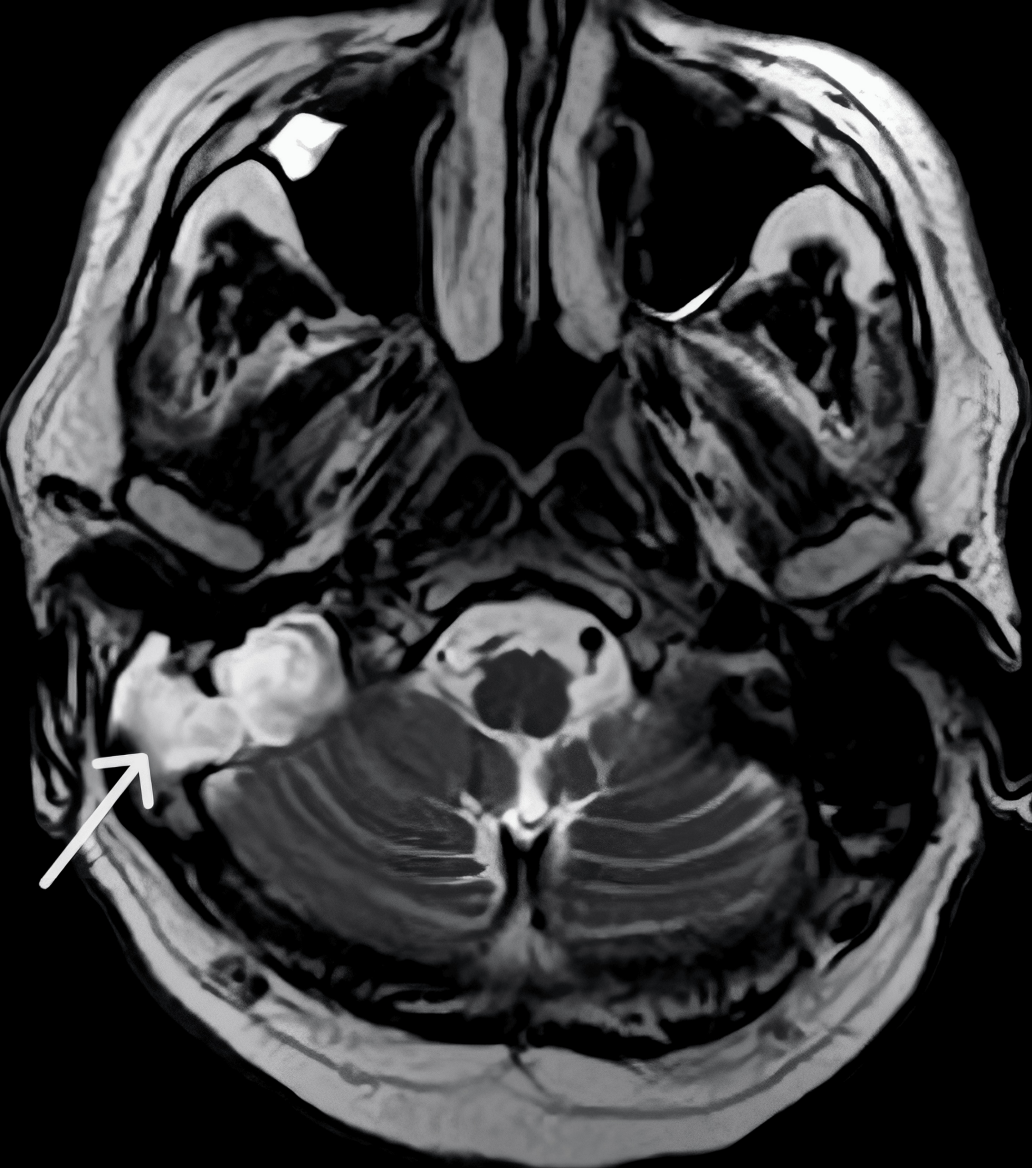
Axial T2-weighted MRI demonstrating a hyperintense lesion in the right temporal bone (white arrow), corresponding to the mastoid region, without evidence of intracranial extension

There was no radiological evidence of intracranial extension or involvement of adjacent vascular structures. MRI demonstrated a high-signal lesion on T2-weighted imaging without intracranial extension (Figure 2).

**Figure 3:**
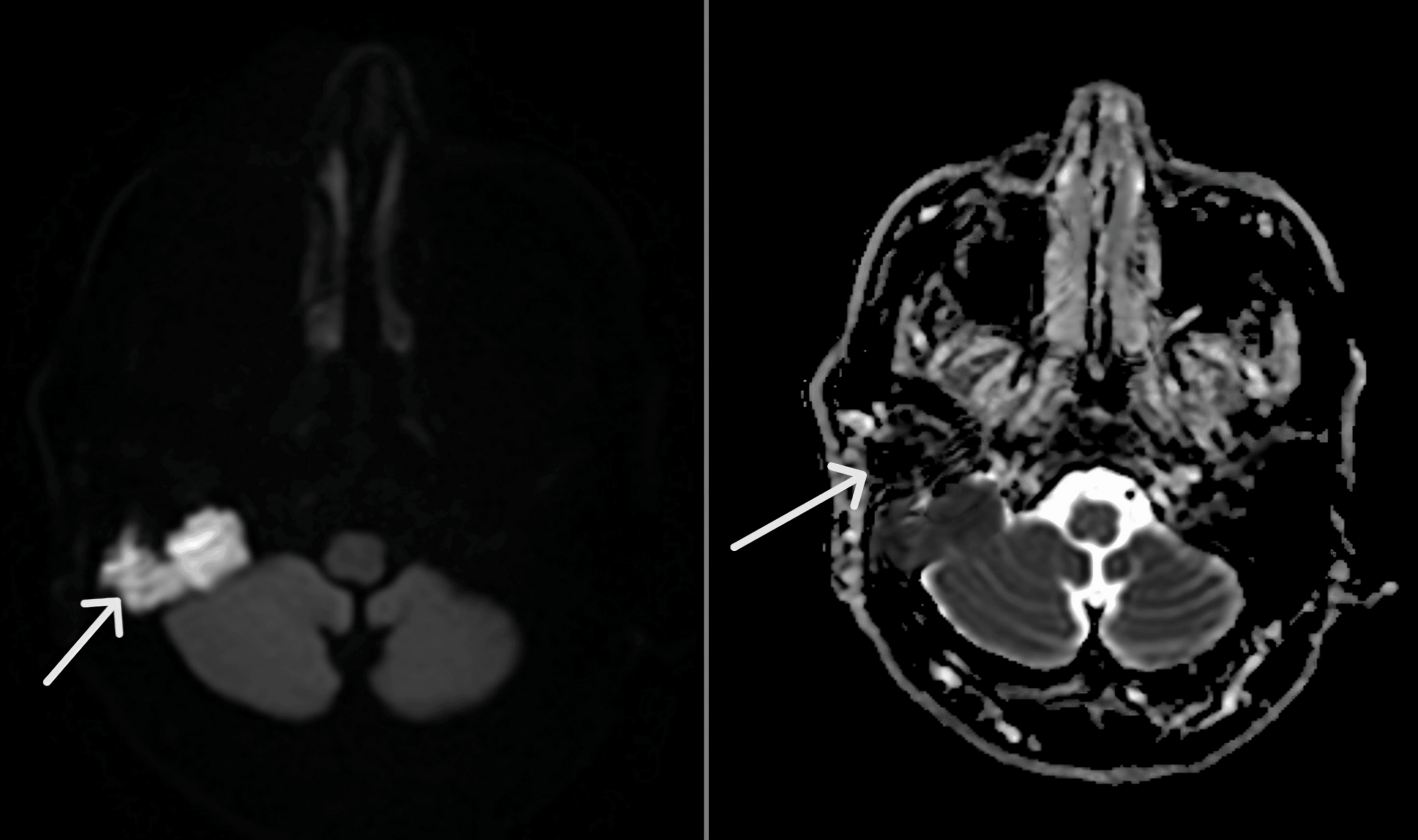
Composite axial MRI showing marked diffusion restriction on diffusion-weighted imaging (left) with corresponding low signal on apparent diffusion coefficient mapping (right), consistent with cholesteatoma (white arrows). The lesion is localized to the right temporal bone/mastoid region

Diffusion-weighted imaging demonstrated marked diffusion restriction with corresponding low signal on apparent diffusion coefficient mapping in the same composite image (Figure 3), consistent with cholesteatoma [5,6].

The case was discussed at the regional skull base multidisciplinary team meeting. Given the extent of disease involving the mastoid, jugular bulb region, and facial nerve canal, subtotal petrosectomy with blind sac closure was recommended to achieve complete disease clearance. Alternative surgical approaches, including canal wall down mastoidectomy, were considered but deemed less suitable due to the extent and location of disease. The patient remains under regular follow-up, with facial nerve function stable at House-Brackmann grade III while awaiting definitive surgical management.

## Discussion

This case highlights the importance of maintaining a high index of suspicion for temporal bone pathology in patients presenting with persistent lower motor neuron facial palsy, particularly in the absence of typical otological symptoms. Cholesteatoma is a well-recognized cause of progressive temporal bone destruction and may lead to complications including ossicular erosion, labyrinthine fistula, intracranial extension, and facial nerve dysfunction [1].

The differential diagnosis for persistent lower motor neuron facial palsy includes idiopathic facial palsy (Bell’s palsy), neoplastic, infectious, and structural causes such as temporal bone lesions, highlighting the importance of imaging in atypical or nonresolving cases.

In this case, the absence of otorrhea, otalgia, or previous chronic ear disease made the diagnosis less clinically apparent. The presence of isolated facial nerve weakness emphasized the need for comprehensive radiological evaluation. Diffusion-weighted MRI played a key role in establishing the diagnosis by demonstrating marked diffusion restriction, a characteristic feature of cholesteatoma that helps differentiate it from other soft tissue lesions of the temporal bone [5,6].

Congenital lesions such as epidermoid cysts or structural abnormalities may present with similar radiological features; however, the pattern of bony erosion, involvement of the mastoid and facial nerve canal, and clinical history in this case were more consistent with an acquired cholesteatoma rather than a congenital lesion.

Surgical management was guided by the extent of disease and involvement of critical anatomical structures, including the facial nerve canal and skull base. A subtotal petrosectomy with blind sac closure was selected to achieve complete disease clearance and minimize the risk of recurrence in the context of extensive disease. Alternative surgical approaches, including canal wall down mastoidectomy, were considered but deemed less suitable due to disease extent and involvement of critical anatomical structures.

Although endoscopic and less invasive techniques may be appropriate in selected cases, more extensive approaches remain necessary in advanced disease to ensure complete eradication and reduce the risk of recurrence.

This case reinforces the importance of early imaging in atypical presentations of facial nerve palsy, the diagnostic value of diffusion-weighted MRI in identifying cholesteatoma, and the need for an individualized surgical approach based on disease extent and anatomical involvement.

## Conclusions

Temporal bone cholesteatoma may present atypically with chronic facial nerve palsy in the absence of classical otological symptoms. Early imaging with CT and diffusion-weighted MRI is essential for diagnosis and assessment of disease extent. This case highlights the importance of considering structural temporal bone pathology in persistent unilateral lower motor neuron facial weakness.

In advanced cases with facial nerve canal involvement and skull base extension, multidisciplinary evaluation is important in determining the most appropriate surgical strategy. Prompt recognition and appropriate referral may help prevent further progression and guide definitive management.
